# Endothelial and Neuronal Nitric Oxide Activate Distinct Pathways on Sympathetic Neurotransmission in Rat Tail and Mesenteric Arteries

**DOI:** 10.1371/journal.pone.0129224

**Published:** 2015-06-15

**Authors:** Joana Beatriz Sousa, Maria Sofia Vieira-Rocha, Silvia M. Arribas, Maria Carmen González, Paula Fresco, Carmen Diniz

**Affiliations:** 1 Laboratory of Pharmacology, Department of Drug Sciences, Faculty of Pharmacy, University of Porto, Porto, Portugal; 2 LAQV/REQUIMTE, University of Porto, Porto, Portugal; 3 Department of Physiology, Faculty of Medicine, Universidad Autónoma de Madrid, Madrid, Spain; Albany Medical College, UNITED STATES

## Abstract

Nitric oxide (NO) seems to contribute to vascular homeostasis regulating neurotransmission. This work aimed at assessing the influence of NO from different sources and respective intracellular pathways on sympathetic neurotransmission, in two vascular beds. Electrically-evoked [^3^H]-noradrenaline release was assessed in rat mesenteric and tail arteries in the presence of NO donors or endothelial/neuronal nitric oxide synthase (NOS) inhibitors. The influence of NO on adenosine-mediated effects was also studied using selective antagonists for adenosine receptors subtypes. Location of neuronal NOS (nNOS) was investigated by immunohistochemistry (with specific antibodies for nNOS and for Schwann cells) and Confocal Microscopy. Results indicated that: 1) in mesenteric arteries, noradrenaline release was reduced by NO donors and it was increased by nNOS inhibitors; the effect of NO donors was only abolished by the adenosine A_1_ receptors antagonist; 2) in tail arteries, noradrenaline release was increased by NO donors and it was reduced by eNOS inhibitors; adenosine receptors antagonists were devoid of effect; 3) confocal microscopy showed nNOS staining in adventitial cells, some co-localized with Schwann cells. nNOS staining and its co-localization with Schwann cells were significantly lower in tail compared to mesenteric arteries. In conclusion, in mesenteric arteries, nNOS, mainly located in Schwann cells, seems to be the main source of NO influencing perivascular sympathetic neurotransmission with an inhibitory effect, mediated by adenosine A_1_ receptors activation. Instead, in tail arteries endothelial NO seems to play a more relevant role and has a facilitatory effect, independent of adenosine receptors activation.

## Introduction

Nitric oxide (NO) contributes to vascular homeostasis [1–3] by modulating the vascular dilator tone and regulating local cell growth. Since NO is an uncharged and highly soluble molecule in hydrophobic environments, it can freely diffuse across biological membranes and signal on vascular cells distant from its site of generation [4]. Therefore, NO can modify vascular smooth muscle tone directly, acting on smooth muscle cells, or indirectly, by modulating sympathetic neurotransmission. In fact, there is evidence demonstrating the influence of NO on sympathetic neurotransmission in various vascular beds, such as mesenteric artery [5–12], pulmonary artery [13–15], heart and coronary arteries [12,16]. There are conflicting data concerning the influence exerted by NO on noradrenaline release: some authors claim that NO inhibits [17,18] whereas other studies showed an increase in noradrenaline release caused by NO [19–21]. However, most of these studies have been performed in heart, brain or urethra and, therefore, information on the direct influence of NO on perivascular sympathetic transmission is not fully understood.

It is conceivable that NO mediated-effects, in addition to the classically accepted activation of intracellular cGMP-dependent pathways [19] can also be related to cGMP-independent pathways, namely by inducing a decrease in mitochondrial respiration, with subsequent adenosine accumulation [22]. Therefore, it is possible that adenosine and its receptors (adenosine receptors) might participate on the modulation of sympathetic neurotransmission exerted by NO. It is worth noting that we have previously demonstrated that adenosine receptors are present in perivascular sympathetic nerves modulating noradrenaline release in mesenteric [23–25] and tail arteries [26–30].

This work aimed to clarify the NO influence on perivascular sympathetic neurotransmission (noradrenaline release), assessing: 1) the source of vascular NO, 2) the intracellular pathways implicated and 3) the potential role of adenosine or its receptors. For this purpose, in the present study, two different vessels were used, mesenteric and tail arteries, which have been extensively used as models for the study of neuromodulation exerted by many substances in the vasculature [5,7,8,31–33] and where we have previously described the presence of adenosine receptors on sympathetic nerves [24,27].

## Materials and Methods

Handling and care of animals were conducted according to the European guidelines (Directive 2010/63/EU) on the protection of animals used for scientific purposes in agreement with the NIH guidelines. This study was carried out in strict accordance with the recommendations in the Guide for the Care and Use of Laboratory Animals of the National Institutes of Health. The protocol was approved by the Committee on the Ethics of Animal Experiments of the University of Porto (Permit Number: 13/11/2013).

### Animals and arterial tissue

Adult male Wistar Kyoto rats (12 weeks old, 270–350 g; Charles River, Barcelona, Spain) were used. Animals were sacrificed using guillotine. Seven arterial segments (5 to 9 mg) were obtained from each tail artery and four arterial segments (4–7 mg) were obtained from each mesenteric artery. Two animals per experiment were used. For each condition, results obtained from 5 to 24 tissue segments were analyzed.

### Chemicals

The following drugs were used: levo-[ring-2,5,6-3H]-noradrenaline, specific activity 41.3 Ci/mmol, was from DuPont NEN (I.L.C., Lisboa, Portugal); Desipramine hydrochloride, Sodium Nitroprusside (SNP), DiethylamineNONOate diethylammonium salt (DEA-NONOate), Nω-Nitro-L-arginine methyl ester hydrochloride (L-NAME), Nω-Propyl-L-arginine hydrochloride and L-NIO dihydrochloride, desipramine hydrochloride, 8-cyclopentyl-1,3-dipropylxanthine (DPCPX), 7-(2-phenylethyl)-5-amino-2-(2-furyl)-pyrazolo-[4,3-e]-1,2,4-triazolo[1,5-c] pyrimidine (SCH 58261), 5-Iodotubericidin (ITU) and Triton X-100 were purchased from Sigma-Aldrich (Sintra, Portugal). The following antibodies were used: mouse monoclonal anti-NOS1 (sc-5302),were purchased from Santa Cruz Biotechnology, Inc., CA, USA; rabbit GFAP polyclonal antibody (18–0063) was purchased from Invitrogen, Life Technologies, SA, Madrid, Spain). The following fluorescent probes were used: Alexa Fluor 488 goat anti-mouse IgG (H+L) antibody, highly cross-adsorbed and Alexa Fluor 647 goat anti-rabbit IgG (H+L) antibody, highly cross-adsorbed (Molecular Probes) secondary fluorescent antibodies (Invitrogen, Life Technologies, SA, Madrid, Spain); vectashield mounting medium with DAPI (Vector Laboratories, UK). Stock solutions were made up in dimethylsulphoxide (DMSO: 0.01% v/v, final concentration) or distilled water and diluted in superfusion medium immediately before use. DMSO was added to the superfusion medium (final concentration 0.01%), in parallel control experiments.

### [^3^H]-Noradrenaline release experiments

[^3^H]-noradrenaline release experiments were carried out as previously described [23,24,27–29]. Briefly, the arterial segments were pre-incubated in 2 ml Krebs-Henseleit solution containing 0.1 μmol/L [^3^H]- noradrenaline (for 60 min at 37°C) and transferred to superfusion chambers, superfused with [^3^H]-noradrenaline-free medium (1 ml.min-1; constant rate: Krebs-Henseleit solution with desipramine 400 nmol/L to inhibit noradrenaline’s neuronal uptake). Up to three periods of electrical stimulation (5 Hz, 100 pulses, 1 ms, 50 mA; Hugo Sachs Elektronik, March-Hugstetten, Germany): two stimulation periods (S_1_ and S_2_) were applied with 30 min intervals, t = 90 min and t = 120 min, respectively. The superfusate was collected each 5 min period from 85 min of superfusion onwards. At the end of the experiments (t = 130 min), tritium was measured in superfusate samples and solubilized arteries (sonicated for 1h with 2.5 ml perchloric acid (0.2 mol/L)) by liquid scintillation spectrometry (LS 6500, Beckman Instruments, Fullerton, USA) after adding 6 ml of a scintillation mixture (OptiPhase ‘Hisafe’ 3, PerkinElmer, I.L.C., Lisboa, Portugal) to each sample. Tissue labelling with [^3^H]-noradrenaline and evaluation of electrically-evoked tritium overflow changes was performed as previously described [23,24].

To study the effects of NO from different sources, experiments were performed in the presence or absence of NOS inhibitors (Nω-Nitro-L-arginine methyl ester hydrochloride, Nω-Propyl-L-arginine hydrochloride and L-NIO dihydrochloride) and donors (SNP and DEA-NONOate), adenosine receptors antagonists (DPCPX and SCH 58621) or the adenosine kinase inhibitor (ITU), which were added after S_1_ and kept until the end of the experiment.

### Laser scanning confocal microscopy (LSCM) experiments

Immunohistochemistry procedures were performed as previously described [24]. From each artery four segments were obtained, immediately placed in cold phosphate buffer solution (PBS; in g/L): NaCl 8.0, Na2HPO4.2H2O 0.77, KCl 0.20, KH2PO4 0.19 (pH 7.2), longitudinally opened and fixed (paraformaldehyde 4% in PBS; 50 min; room temperature, RT). After two 15 min washing cycles with PBS-T (PBS with 0.3% of Triton X-100), each arterial segment was incubated with the primary antibodies against: nNOS, (mouse monoclonal anti-NOS1; 1:200 dilution, overnight, 4°C) and GFAP (rabbit polyclonal anti-glial fibrillary acidic protein, 1:200 dilution, overnight, 4°C). After a PBS (4x15min) washout period, tissues were incubated with Alexa 488 anti-mouse and Alexa 647 anti-rabbit fluorescent secondary antibodies (1:1000 dilution, 1h, RT). Negative controls were performed by omitting primary antibodies. After two PBS washing cycles, tissue preparations were mounted with antifading agent (vectashield mounting medium with DAPI, Vector Laboratories, UK), with the adventitial side facing up. Preparations were visualized with a Leica SP5 laser scanning confocal microscopy system (Leica Microsystems, Germany) fitted with an inverted microscope (x63 oil immersion lens). Stacks of 1-μm-thick serial optical images were captured from the entire adventitial layer, which was identified by the shape and orientation of the nuclei. Image acquisition was performed always under the same laser power, brightness and contrast conditions. Serial images from 3 different regions of the adventitia layer were acquired from each mesenteric and tail artery at 360 nm Ex/ 460 nm Em (for location of cell nuclei), at 488 nm Ex/525 nm Em (location of nNOS containing cells) and at 633 nm Ex/665 nm Em (location of Schwann cells) wavelengths. The resulting images were reconstructed separately for each wavelength with the Leica SP5 laser scanning confocal microscopy system (Leica Microsystems, Germany).

### Data Analysis

#### Measurement of drug effects on electrically-evoked tritium overflow

Electrically-evoked tritium overflow from artery segments incubated with [^3^H]-noradrenaline has been shown to reflect action potential-evoked neuronal noradrenaline release and drug-induced changes in evoked tritium overflow are assumed to reflect changes in neuronal noradrenaline release. Effects of drugs added after S_1_ on electrically-evoked tritium overflow were evaluated as ratios of the overflow elicited by S_2_ and the overflow elicited by S_1_ (S_2_/S_1_). S_2_/S_1_ ratios obtained in individual experiments in which a test compound A was added after S_1_ were calculated as a percentage of the respective mean ratio in the appropriate control group (solvent instead of A) [25–27,34–36].

#### Laser Scanning Confocal Microscopy image quantification

Quantitative analysis of confocal z-stacks images was performed using an image analysis software (PAQI, CEMUP, OPorto, Portugal). Briefly, a sequential routine was designed and developed to analyze each fluorescent signal used. PAQI software measured the surface area and intensity of immunofluorescence of the antibody against neuronal NOS isoform, the surface area and intensity of immunofluorescence of the antibody against GFAP and determined the surface area of attachment of the antibodies anti-nNOS and anti-GFAP.

#### Statistics

Results are expressed as mean±s.e.m. and *n* denotes the number of animals used with triplicate tissue preparations. Differences of means were compared for significance using one-way ANOVA and the *post-hoc* Holm-Sidak’s multicomparisons test. A P value lower than 0.05, 0.01 or 0.001 was considered to denote statistically significant differences.

## Results

**Fig 1:**
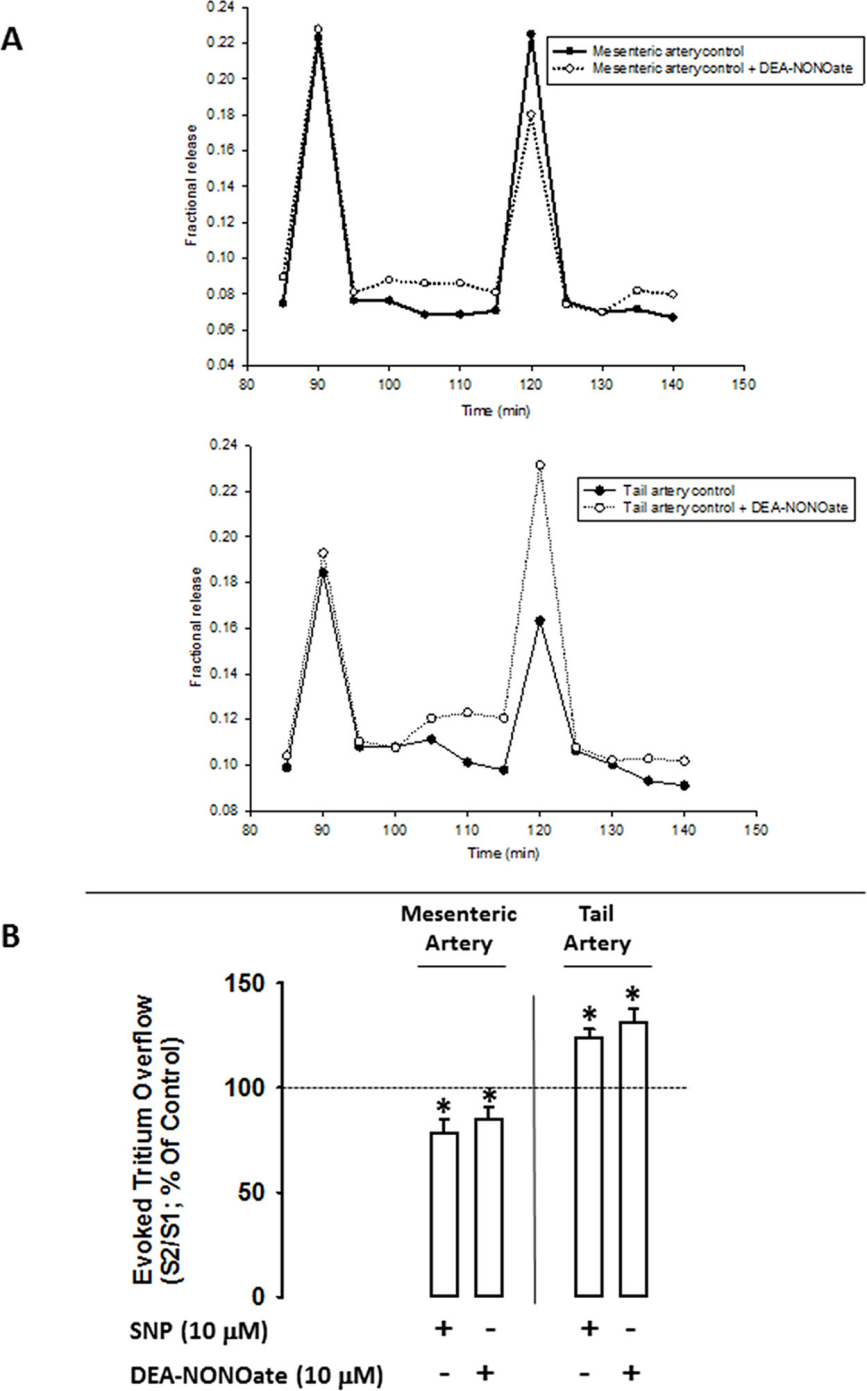
Panel A: time course of fractional tritium release from mesenteric and tail artery controls (filled circles) and from mesenteric and tail arteries with the NO donor, DEA-NONOate (open circles) taken from a typical experiment. Each line represents the outflow of tritium from a single superfusion chamber. After pre-incubation with [^3^H]-noradrenaline, tissues were superfused with [^3^H]-noradrenaline free medium containing desipramine (400 nM). Drugs were added immediately after S_1_ and kept until the end of the experiment. Tritium outflow (ordinates) is expressed as a percentage of the total radioactivity present in the tissue at the beginning of the collection period and was measured in samples collected every 5 min. Artery segments were stimulated twice (S_1_, S_2_) using 100 pulses /5Hz, 1 ms, 50 mA. **Panel B:** Influence of nitric oxide donors, SNP (10μM) and DEA-NONOate (10μM), on the modulation of electrically evoked tritium overflow in tail and mesenteric arteries. Arteries were electrically stimulated (S_1_-S_2_: 100 pulses, 5 Hz, 1 ms, 50 mA). Drugs were added immediately after S_1_ and kept until the end of the experiment. *Ordinates*: S_2_/S_1_ values obtained in individual tissue preparations, expressed as a percentage of the appropriate S_2_/S_1_ control value. Values are mean±s.e.m. from n = 4–6. Significant differences from solvent: *P<0.05 (one-way ANOVA followed by *post-hoc* Holm-Sidak’s multicomparisons *t*-test).

The fractional rate of basal tritium outflow (b_1_), electrically-evoked tritium overflow (S_1_) and S_2_/S_1_ ratios of mesenteric and tail arteries are shown in Table 1 and Fig 1.

**Table 1:** Basal tritium outflow (b_1_), electrically evoked tritium overflow (S_1_) and S_2_/S_1_ ratios from mesenteric and tail arteries.

	Basal Outflow (b_1_) (fractional rate of outflow; min^-1^)	Evoked Overflow (S_1_) (% of tissue tritium content)	S_2_/S_1_	*n*
***Mesenteric artery***				
Solvent	0.070±0.008	0.229±0.037	0.9801±0.048	6
DEA-NONOate	0.068±0.008	0.227±0.037	0.8082±0.063 *	4
***Tail artery***				
Solvent	0.077±0.003	0.185±0.015	1.0275±0.081	6
DEA-NONOate	0.075±0.009	0.193±0.015	1.2665±0.067 *	4

Tissue preparations of mesenteric and caudal arteries were pre-incubated with [^3^H]-noradrenaline for 40 min. After pre-incubation with [^3^H]-noradrenaline, tissues were superfused with [^3^H]-noradrenaline free medium containing desipramine (400 nM). Tissues were stimulated twice at 30-min intervals (S_1_-S_2_; 100 pulses, 5 Hz, 1 ms, 50 mA): b_1_ refers to the 5-min period immediately before S_1_. The electrically-evoked tritium overflow was calculated by subtracting the estimated basal outflow from total outflow observed during and in the 25-min period subsequent to S_1_ and expressed as a percentage of the tissue tritium content at the onset of stimulation. Values presented are means±SEM and *n* denotes the number of tissue preparations. Means were compared for significance using one-way ANOVA, followed by *post-hoc* Holm-Sidak´s multicomparisons *t*-test. Significant differences from the respective solvent: *P<0.050.

Basal outflow and electrically-evoked tritium overflow remained constant throughout the control experiments, with b_n_/b_1_ and S_n_/S_1_ values close to unity. Electrically-evoked tritium overflow (S_1_) was similar in mesenteric and tail arteries. However, in the presence of NO donors, DEA-NONOate (10 μM) or SNP (10 μM), opposite effects in the S_2_ electrically-evoked tritium overflow were observed: in mesenteric arteries, NO donors caused an inhibition whereas in tail arteries these compounds induced facilitation, as depicted in Fig 1.

**Fig 2:**
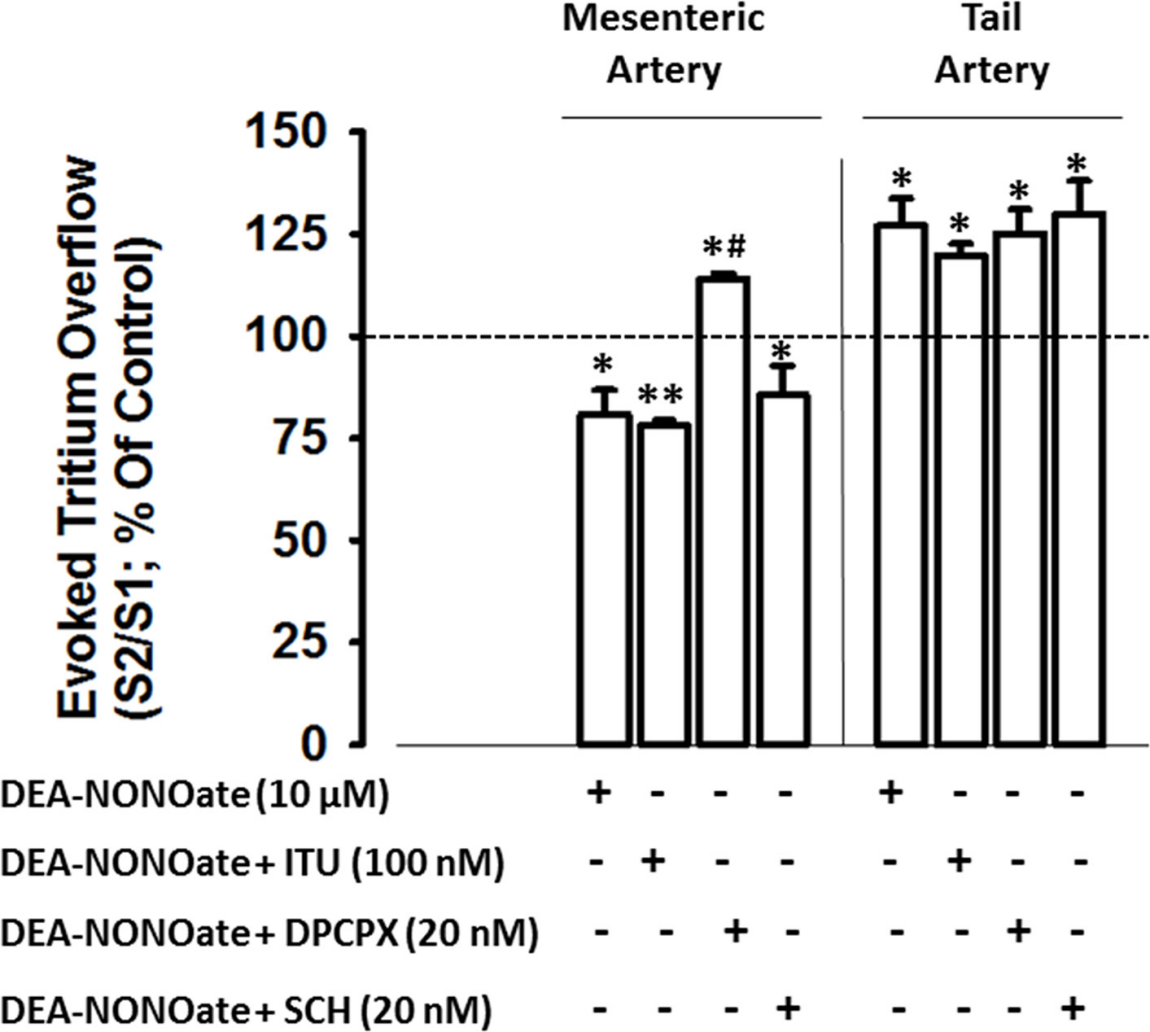
Influence of the adenosine kinase inhibitor (ITU), the adenosine A_1_ receptors antagonist (DPCPX) and the adenosine A_2A_ receptors antagonist (SCH 58261) in the nitric oxide donor DEA-NONOate (10μM) induced modulation of electrically evoked tritium overflow, in mesenteric and tail arteries. Arteries were electrically stimulated (S_1_-S_2_: 100 pulses, 5 Hz, 1 ms, 50 mA). Drugs were added immediately after S_1_ and kept until the end of the experiment. *Ordinates*: S_2_/S_1_ values obtained in individual tissue preparations, expressed as a percentage of the appropriate S_2_/S_1_ control value. Values are mean±s.e.m. from n = 4–6. Significant differences from the appropriate control: *P<0.05; **P<0.01 and from DEA-NONOate: ^#^P<0.001 (one-way ANOVA followed by *post-hoc* Holm-Sidak’s multicomparisons *t*-test).

In the mesenteric artery, inhibition of electrically-evoked tritium overflow induced by DEA/NONOate was abolished by the selective adenosine A_1_ receptors antagonist, DPCPX (20 nM), and was not altered by the selective adenosine A_2A_ receptors antagonist, SCH 58261 (20 nM), or by the adenosine kinase inhibitor, ITU (100 nM; Fig 2). In the tail artery, however, neither adenosine receptors antagonists (DPCPX and SCH 58261) nor the adenosine kinase inhibitor, ITU, affected facilitation of electrically-evoked tritium overflow elicited by NO. Adenosine receptor antagonists did not influence tritium overflow as depicted in Table 2.

**Table 2:** Adenosine receptors antagonists mediated tritium overflow from mesenteric and tail arteries (S_2_/S_1_ ratios).

	S_2_/S_1_ (% of solvent)	*n*
***Mesenteric artery***		
**DPCPX**	101.41 ± 2.67	4
**SCH 58261**	106.04 ± 3.08	8
***Tail artery***		
**DPCPX**	99.46 ± 3.81	6
**SCH 58261**	98.70 ± 7.99	10

Tissue preparations of mesenteric and caudal arteries were pre-incubated with [^3^H]-noradrenaline for 40 min. After pre-incubation with [^3^H]-noradrenaline, tissues were superfused with [^3^H]-noradrenaline free medium containing desipramine (400 nM). Tissues were stimulated twice at 30-min intervals (S_1_-S_2_; 100 pulses, 5 Hz, 1 ms, 50 mA). The electrically-evoked tritium overflow was calculated by subtracting the estimated basal outflow from total outflow observed during and in the 25-min period subsequent to S_1_ and expressed as a percentage of the tissue tritium content at the onset of stimulation. Values presented are means ± SEM and *n* denotes the number of tissue preparations. Means were compared for significance using one-way ANOVA, followed by *post-hoc* Holm-Sidak´s multicomparisons *t*-test. No significant differences from the respective solvent were observed.

**Fig 3:**
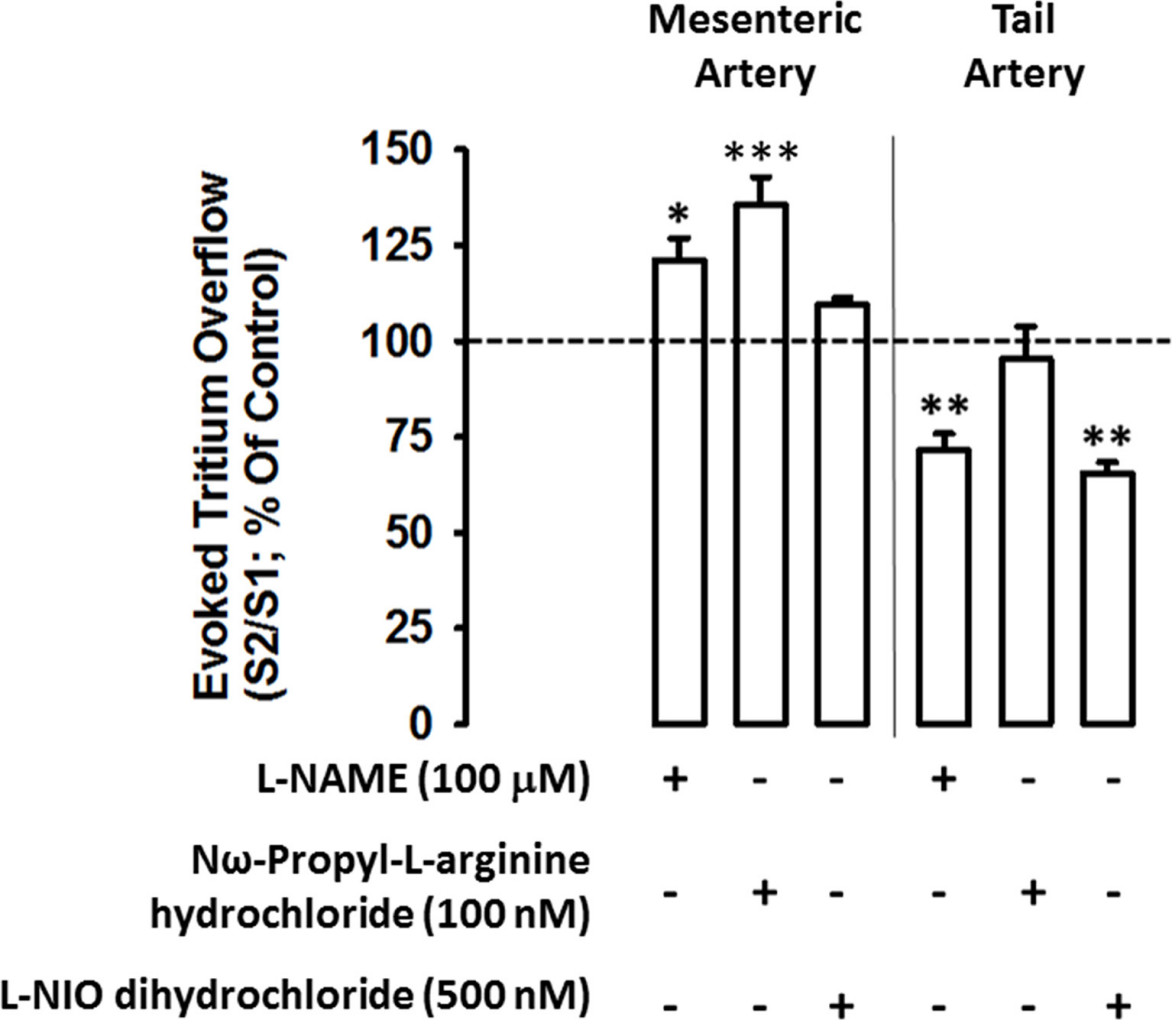
Influence of nitric oxide inhibitors on the modulation of electrically- evoked tritium overflow in tail and mesenteric arteries: interaction with nitric oxide inhibitors L-NAME (100 μM, a non-selective nNOS inhibitor), Nω-Propyl-L-arginine hydrochloride (100 nM, a selective nNOS inhibitor) and L-NIO dihydrochloride (500 nM, a selective eNOS inhibitor). Arteries were electrically stimulated (S_1_-S_2_: 100 pulses, 5 Hz, 1 ms, 50 mA). Drugs were added immediately after S_1_ and kept until the end of the experiment. *Ordinates*: S_2_/S_1_ values obtained in individual tissue preparations, expressed as a percentage of the appropriate S_2_/S_1_ control value. Values are mean±s.e.m. from n = 4–6. Significant differences from the appropriate control: *P<0.05; **P<0.01; ***P<0.001 (one-way ANOVA followed by *post-hoc* Holm-Sidak’s multicomparisons *t*-test).

In mesenteric artery, L-NAME (a non-selective NOS inhibitor) and Nω-Propyl-L-arginine hydrochloride (a selective nNOS inhibitor) facilitated electrically-evoked tritium overflow up to 21% and 35%, respectively (Fig 3). However, L-NIO dihydrochloride, a selective eNOS inhibitor, was devoid of effect. In tail artery, however, the effect elicited by NOS inhibitors on the electrically-evoked tritium overflow presented an opposite profile: L-NAME and L-NIO dihydrochloride inhibited electrically-evoked tritium overflow (in approximately 30%), an effect compatible with the facilitatory role of NO donors described above in this artery, whereas the nNOS inhibitor, failed to change tritium release (Fig 3).

Additional experiments were performed with endothelium removal (endothelium was mechanically removed from arteries with a stainless steel wire) in order to confirm the existence of the two NO sources: endothelial or neuronal. In fact, endothelium removal in tail artery reverted L-NAME and L-NIO dihydrochloride mediated effects on the electrically-evoked tritium overflow (S2/S1, % of appropriate control, were 97,16% ± 8,33; n = 6 and 109,56% ± 13,03; n = 5, for L-NAME and L-NIO dihydrochloride, respectively). Moreover, for mesenteric artery, endothelium removal did not modified L-NAME mediated effects when compared with results obtained in intact arteries (S2/S1, % of appropriate control, was 117,51% ± 2,67; n = 4, for L-NAME). Taking into consideration that L-NAME is a non-specific NOS inhibitor and have the ability to inhibit both endothelial and neuronal NOS and, since L-NAME data, in denuded and intact arteries, present the same effect on electrically-evoked tritium overflow, the putative involvement of endothelial NO in altering neurotransmission can be discarded.

**Fig 4:**
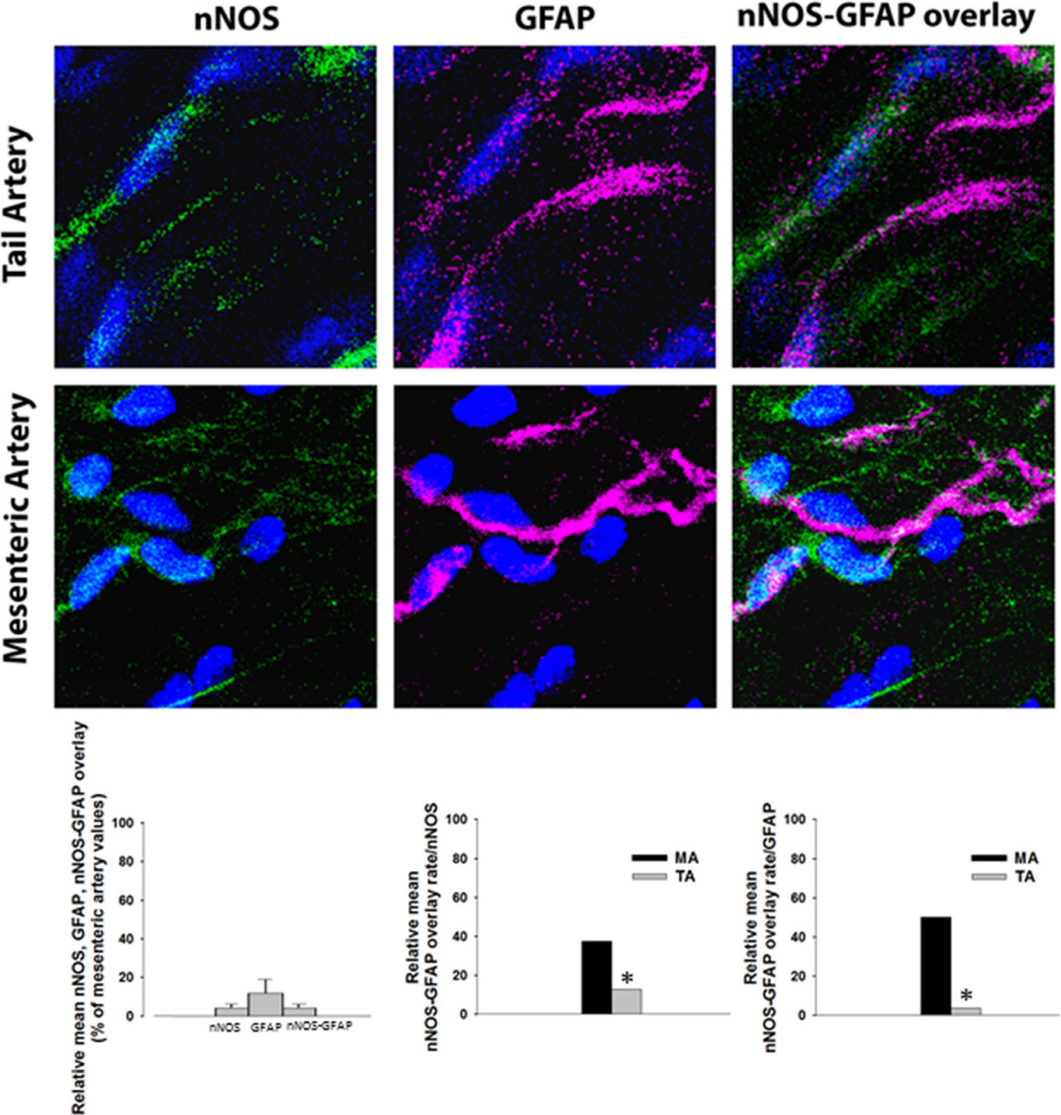
nNOS in Schwann cells in the adventitia of tail and mesenteric arteries. (A) Representative reconstructions of the adventitia from tail and mesenteric arteries. Images were captured with a confocal microscope (Leica SP5 LSCM system fitted with an inverted microscope (x63 oil immersion lens). Stacks of 1μm-thick serial optical images were captured from the entire adventitial layer and reconstructed by software. Arteries were stained for nNOS (a primary mouse monoclonal anti-NOS1 and a species specific secondary Alexa 488 antibody: green), GFAP (a primary rabbit anti-GFAP polyclonal antibody and a species specific secondary Alexa 647 antibody: magenta) and DAPI (nuclear stain, blue). Filled arrows evidence nNOS and GFAP overlaid immunoreactivities; open arrows evidence nNOS immunoreactivities in cells other than Schwann cells, present in adventitia. (B) Relative means of nNOS, GFAP and nNOS-GFAP overlay expressed as percentage of mesenteric artery values. (C) Mean percentage of overlay rate with GFAP and (D) mean percentage of overlay rate with nNOS are depicted. Values are mean±s.e.m. from n = 3–4. Significant differences from mesenteric artery: *P<0.05. Scale bar = 20 μm.

Results indicate distinct NO modulatory roles in the two vessels in study which can be related with differences in the NO source. To confirm this later possibility, the immunoreactivity for nNOS, in the mesenteric and tail arteries, was studied (Fig 4A). We focused our attention to he vascular adventitia layer since our goal was to analyse the vascular sympathetic neurotransmission, an event that occurs in the sympathetic nerves spread in the adventitia layer. LSCM images from the adventitial layers, of both mesenteric and tail arteries, exhibited nNOS isoform immunoreactivities (Fig 4A), but quantitative analysis evidenced that the relative amount of nNOS immunoreactivity present in tail arteries is lower (up to 27% lower) than that exhibited in mesenteric arteries (Fig 4B). These results together with functional data (Fig 3) reveal and support a differential role of nNOS in the tail and mesenteric arteries.

We have previously demonstrated that sympathetic nerves are surrounded by Schwann cells (anti-GFAP-immunoreactivity) [25]. The putative presence of nNOS isoform in Schwann cells was challenged. Data form LSCM images evidenced the occurrence of nNOS and GFAP overlaid immunoreactivities (Fig 4A; depicted by filled arrows). Nevertheless, nNOS and GFAP overlaid immunoreactivities are markedly lower in tail comparatively to mesenteric arteries (after data normalization by total GFAP immunoreactivity): 50% of Schwann cells exhibit nNOS immunoreactivity in mesenteric arteries *versus* 4% observed in tail arteries. Similar results were also observed in Fig 4D (upon normalization of nNOS-GFAP overlay with total nNOS immunostaining). Indeed, nNOS and GFAP staining overlay might indicate that these proteins can be located on the same cellular structure. Nonetheless, nNOS immunoreactivity was also observed in cells present in adventitia, other than Schwann cells, in both mesenteric and tail artery images (depicted by open arrows): 62.5% in mesenteric and 88% in tail artery images (Fig 4, A e D).

Taken together, these morphological data support the occurrence of differences on nNOS isoform presence in mesenteric and tail arteries and reveal an important role for Schwann cells as an NO source, in mesenteric arteries.

## Discussion

The present results demonstrate that the NO modulatory role on sympathetic neurotransmission and the different contribution of NO from neuronal and endothelial sources are dependent on the vascular bed. In mesenteric arteries nNOS, mainly localized in Schwann cells, seems to be the main source of NO influencing perivascular sympathetic neurotransmission, while in tail arteries the endothelium seems to play the most relevant role. Intracellular signalling cascades and adenosine receptors involved in the two vessels are also different.

In mesenteric arteries we found that an increase in NO availability, *via* addition of NO donors, reduced noradrenaline release in the synaptic cleft. As a consequence, a reduction in the vasoconstrictor effect mediated by activation of smooth muscle α_1_-adrenoceptors will occur. These results are in line with previous studies that reported an altered vascular reactivity induced by NO at the sympathetic neuroeffector junction, ascribed to the deactivation of the vasoconstrictor, noradrenaline in the rat mesenteric bed [37]. However, in tail arteries NO mediated an opposite effect: an increase of noradrenaline release levels. Taken together, these data seem to indicate a different neuromodulatory role of NO in the neurovascular junction of mesenteric and tail arteries, most likely, due to the activation of different pathways.

In addition to the well characterized classical mechanism by which NO mediates its effects, *via* sGC-cGMP-PKG [38–41], results obtained in the current study, suggest that, in mesenteric arteries, inhibition of sympathetic transmission by NO seems to activate an alternative signaling pathway and/or the involvement of different NOS isoforms. Increasing evidence suggest that NO may also signal through a cGMP independent pathway: NO inhibits the Krebs cycle [42], and inhibits complex I and IV of the mitochondrial respiratory chain [43,44] compromising the mitochondrial function in neurons [45] leading to ATP hydrolysis and subsequent accumulation of adenosine [46,47] that, in turn, can signal through activation of high affinity adenosine receptors (A_1_ and A_2A_ subtypes) or low affinity adenosine receptors (A_2B_ and A_3_ subtypes). Indeed, the inhibition of noradrenaline release induced by NO seems to be mediated by activation (by adenosine) of the adenosine A_1_ receptors subtype since the inhibition of noradrenaline release mediated by NO donors was prevented by the adenosine A_1_ receptors antagonist (DPCPX) but was not altered by the adenosine A_2A_ receptors antagonist (SCH 58261). Similar NO-mediated mechanisms have also been described to occur in several central nervous system tissues, such as the hippocampus [48–50] or the nucleus tractus solitarius [51].

It is well established that adenosine availability in the extracellular space may depend on nucleoside transporters and on the activity rate of adenosine kinase [52–54], as well as on the neuronal exocytotic ATP release [55–57]. In mesenteric arteries, we have demonstrated previously that exocytosis (induced by electrical field stimulation) did not modify the amount of adenosine available at the extracellular space [25]. Therefore, adenosine accumulation in the present conditions could result from the transport of adenosine *via* nucleoside transporters or from adenosine kinase inhibition (adenosine kinase is inhibited by adenosine itself). The first hypothesis was previously demonstrated by our group [25] whereas the later possibility was ruled out in the present work, since ITU (an adenosine kinase inhibitor) was devoid of effect upon the NO-induced (with NO donors) inhibition of noradrenaline release.

On the other hand, in mesenteric arteries, selective NOS isoform inhibitors also differentially influenced noradrenaline release: nNOS inhibitors increased noradrenaline release, an effect in agreement with results from previous studies in rat [7,9,10,37,58] and rabbit [59] which described NO as a mediator able to reduce noradrenaline release. Instead, the eNOS inhibitor showed no significant effect. Moreover, the non-selective NOS inhibitor, which inhibits both isoforms (nNOS and eNOS), modified noradrenaline release in mesenteric arteries in a similar way to that observed for the nNOS inhibitor. These results suggest that, in mesenteric arteries, only nNOS contributes to NO generation that, ultimately, will lead to the decrease in noradrenaline release. NO generation can be ascribed two both oxygenase and reductase domains of NO synthase. L-NAME, described to be unable to modify the activity of the reductase domain [60], did not completely revert the endogenous NO mediated effects on noradrenaline release whereas the nNOS inhibitor completely reverted it.

Similar experiments were carried out in the tail artery but, in this vessel, the NO-mediated effects (increase of noradrenaline release) are due to NO generated only by the eNOS isoform. The selective eNOS inhibitor and of the non-selective NOS inhibitor, caused a decrease in noradrenaline release but the selective nNOS inhibitor failed to modify noradrenaline release in tail arteries. Therefore, NO generated by eNOS isoform, in tail arteries, increases noradrenaline release, as previously suggested to occur in mice kidneys, where the presynaptic active NO seemed to be exclusively produced by the eNOS isoform [61].

It is accepted that NOS produces NO as result of the oxygenase and reductase domains activities which are responsible for the conversion of L-arginine to L-citrulline plus NO and of the conversion of nitrites to NO, respectively [62]. This later reaction was previously shown to be not affected by L-NAME [60]. In tail artery, the inhibition promoted by eNOS inhibitors (L-NAME and L-NIO dihydrochloride) completely reverted the effects mediated by NO on sympathetic transmission (Fig 3). These results indicate that, in tail arteries, the major contribution for NO production seems to be ascribed to the eNOS oxygenase domain.

In tail arteries, the enhancement of noradrenaline release (induced by NO) is, most likely, due to the activation of signaling cascades different from those observed in mesenteric arteries. In fact, data showed that the effects mediated by NO donors in sympathetic transmission were not altered by the adenosine receptors antagonists studied (DPCPX and SCH 58261), nor the adenosine kinase inhibitor, (ITU). These data do not support the occurrence of an adenosine accumulation evoked by NO mediated effects on mitochondrial respiration [47], which are known to require higher NO amounts [49]. This can be explained by a residual activity of the eNOS reductase domain in tail arteries. Taken together, these data strongly suggests that this is not the predominant pathway by which NO mediate its effects, in tail artery. Classically, it is accepted that NO mediate its effects by activating guanylyl cyclase to generate cGMP. cGMP and cGMP-dependent protein kinase are capable of modulating membrane potential and ion channels [19,63]. Moreover, a cGMP-mediated enhancement of Ca^2+^ channel currents in sympathetic neurons has also been reported [64]. It is, therefore, possible that the facilitation of noradrenaline release induced by NO can be ascribed to cGMP-mediated changes in activated voltage-dependent Ca^2+^ channels [19]. Moreover, data obtained in denuded arteries constitute an additional support for hypothesis of the existence of distinct NO sources (confirmed by the selective NOS inhibitors mediated effects) that will be responsible for activation of distinct pathways on sympathetic neurotransmission in rat tail and mesenteric arteries.

Confocal microscopy studies showed the presence of nNOS isoform in the adventitia layer of both mesenteric and tail arteries. These data agree well with those from a study that described the presence of nNOS isoform in mesenteric arteries [58]. Confocal microscopy data are in agreement with the absence of nNOS effect on sympathetic neurotransmission found in the tail artery, since a considerably lower amount of this isoform (27% of reduction) was observed in tail arteries, comparatively to mesenteric arteries. Our data also show, for the first time, that nNOS is distributed in two main locations: one expressed in Schwann cells and another, more abundant in other cells, about 63% and 88% in the adventitia of mesenteric and tail arteries, respectively. Therefore, it is conceivable that nNOS, expressed in Schwann cells, might be producing NO that would be causing the inhibition of noradrenaline release from sympathetic nerves. Our findings are in agreement with this possibility, since the relative amount of nNOS expressed in Schwann cells in the two arteries is markedly different: 50% in mesenteric *versus* 4% in tail arteries. These data also correlates with the functional results obtained, showing a lack of neuromodulatory role of nNOS in tail arteries whereas in mesenteric arteries nNOS contributed to reduce noradrenaline release up to 33% (Fig 3).

In summary, the present results suggest that the NO modulatory role on sympathetic neurotransmission differs in the mesenteric and tail arteries depending on the NO source, eNOS and/or nNOS, involved in its production. Moreover, the signalling cascades induced by the NO available in each artery are also distinct in the two vessels: in mesenteric artery, NO leads to adenosine accumulation which activates adenosine A_1_ receptors causing an inhibition of sympathetic transmission whereas, in tail arteries, this *via* is not the dominant one, and instead, the activation of guanylyl cyclase–cGMP- cGMP-dependent protein kinase- voltage-dependent Ca^2+^ channels is most likely to occur. Additionally, this work revealed that the location of the nNOS isoform may also be crucial to the differences in the neuromodulation exerted by NO in the two arteries studied, since the nNOS isoform present in Schwann cells seem to be the main source of NO to perivascular sympathetic nerves.
